# Spastin's Microtubule-Binding Properties and Comparison to Katanin

**DOI:** 10.1371/journal.pone.0050161

**Published:** 2012-12-13

**Authors:** Thomas Eckert, Doan Tuong-Van Le, Susanne Link, Lena Friedmann, Günther Woehlke

**Affiliations:** Department of Physics E22 (Biophysics), Technische Universität München, Garching, Germany; Griffith University, Australia

## Abstract

Spastin and katanin are ring-shaped hexameric AAA ATPases that sever microtubules, and thus crucially depend on a physical interaction with microtubules. For the first time, we report here the microtubule binding properties of spastin at the single-molecule level, and compare them to katanin. Microscopic fluorescence assays showed that human spastin bound to microtubules by ionic interactions, and diffused along microtubules with a diffusion coefficient comparable to katanin. The microscopic measurement of landing and dissociation rates demonstrated the ionic character of the interaction, which could be mapped to a patch of three lysine residues outside of the catalytic domain of human spastin. This motif is not conserved in *Drosophila* spastin or katanin, which also bound by non-catalytic parts of the protein. The binding affinities of spastin and katanin were nucleotide-sensitive, with the lowest affinities under ADP,, the highest under ATP-γS conditions. These changes correlated with the formation of higher oligomeric states, as shown in biochemical experiments and electron microscopic images. Vice versa, the artificial dimerization of human spastin by addition of a coiled coil led to a constitutively active enzyme. These observations suggest that dimer formation is a crucial step in the formation of the active complex, and thus the severing process by spastin.

## Introduction

The microtubule cytoskeleton displays an amazing degree of plasticity. There are several mechanisms responsible for the dynamic properties of microtubules: depolymerization from one or both ends, as well as chopping the filament [1]–[3]. The latter process has been termed ‘severing’, and occurs in many eukaryotic phyla. Three related families of severing enzymes have been found: katanin, fidgetin and spastin, all of which belong to the superfamily of AAA ATPases [4]. They share homology in their C-terminal AAA domains, but are unrelated in their N-terminal parts. Animal models and cellular studies suggest that they are involved in different cell and tissue-specific processes (http://www.informatics.jax.org/phenotypes.shtml). The sequence homology of their catalytic domains, however, indicates that these types of enzymes may function according to a similar biomechanical mechanism. Evidently, one of the crucial steps of the severing process is the interaction with the microtubule. As other AAA ATPases, severing enzymes have been reported to be active in the form of homo-hexameric rings [5]–[8]. It has been hypothesized that the C-termini of α or β tubulin are threaded through the central pores of hexameric katanin or spastin rings [6], [7]. This mechanism was proposed by analogy to ClpA, ClpB, and related AAA ATPases, which are parts of proteolytic complexes, or of molecular machines that dissolve protein aggregates [9]. These enzymes recognize characteristic sequence motifs in their substrate molecules, and subsequently pull the polypeptide chain through the central pore.

If severing enzymes used the same mechanisms, they would be expected to lie flat on the surface of the microtubule lattice [6]. Moreover, one might expect a conserved filament-binding motif in severing enzymes that serves as an anchor point, similar to kinesin or myosin motor proteins. Finally, one might suspect severing enzymes to interact with the isolated C-terminal peptide of either α or β tubulin [6]. None of these naïve expectations has been fully met, though, possibly because of incomplete knowledge or technical reasons. For example, White et al. observed binding to a tubulin peptide that, however, had to be significantly larger than the ‘E-hook’ [6]. Possibly, other peptides used in this and other studies lacked additional essential motifs, or suffered from the lack of post-translational modifications. The problem of identifying the orientation of severing enzymes bound to microtubules is that electron microscopic imaging is prone to fixation artifacts. Finally, identification of the microtubule binding site by mutational analysis is complicated by the hexamerization process involved [7]. Hence, it is still not clear how the severing works mechanistically, and how it depends on microtubule-binding. To get deeper insight into the severing mechanism, we compare here the microtubule interaction of spastin and katanin p60.

**Figure 1:**
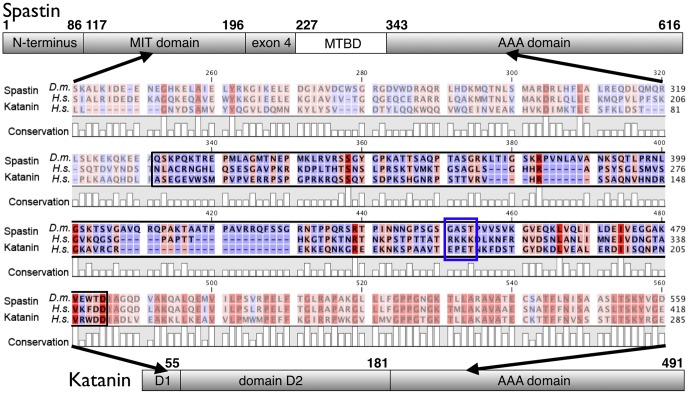
Domain Organization and Alignment of Spastin and Katanin. The figure shows a sequence alignment of domains of *Drosophila* and human spastin, as well as human katanin. The black contour highlights the position of the human spastin linker, residues with a high degree of conservation are red, residues with low degree are blue. The location of the highly basic patch in human spastin is boxed in blue. Above the alignment, the location of the sequence in the context of human spastin is indicated, below the location in human katanin. For details, see text.

Katanin was the first microtubule-severing enzyme identified [10]. It's microtubule binding characteristics has been studied recently using NMR and biochemical evidence, as well as single-molecule fluorescence microscopy [11], [12]. Binding seemed to occur in an oligomeric state, and to involve diffusion along the microtubule. We are not aware of any publications characterizing the nucleotide-dependence of the katanin-microtubule complex by biochemical means. Spastin's microtubule binding activity has been mapped to a region of 59 amino acid residues N-terminal of the catalytic AAA domain, and has been termed microtubule-binding domain (MTBD; Figure 1; [6]). The situation is convoluted, though, because the human and the *Drosophila* ortholog differ greatly in their non-AAA domains (Figure 1; [8]). *Drosophila* spastin requires a combination of parts from two domains for microtubule binding [7]. Because of its clinical relevance and for simplicity, we use here human spastin as the prototype, and compare the aligned region of *Drosophila* to the human spastin microtubule-binding region.

## Materials and Methods

### Buffers

Buffers used in this study are defined as: BRB80 (80 mM Pipes·KOH, pH 6.8, 2 mM MgCl2, 3 mM Mg·acetate, 0.5 mM EGTA); buffer 1 (50 mM HEPES·KOH, pH 7.4, 150 mM NaCl, 5 mM MgCl2, 5% glycerole (v/v) and 1 U/ml lysozyme); buffer 2 (50 mM HEPES·KOH, pH 7.4, 300 mM NaCl, 5 mM MgCl2, 5% glycerole (v/v)); buffer 3 (50 mM HEPES·KOH, pH 7.4, 300 mM NaCl, 5 mM MgCl2, 5% glycerole (v/v), 50 µM ATP and protease inhibitor); buffer 4 (50 mM HEPES·KOH, pH 7.4, 300 mM NaCl, 5 mM MgCl2, 5% glycerole (v/v), 50 µM ATP, 100 µg/ml FLAG-peptide); buffer 5 (50 mM HEPES·KOH, pH 7.4, 5 mM MgCl2, 5% glycerole (v/v)).

### Molecular biology and protein methods

The constructs used were based on a cDNA clone of human spastin (gift from Dr. C. Beetz, Institut fur Klinische Chemie und Laboratoriumsdiagnostik, Universitatsklinikum Jena, Germany). N-terminal GFP fusions (GFP-Δ227 HsSpastin) were generated from the Δ227 human spastin GST expression vector used previously [8]. The protein was expressed in *E. coli* BL21(RIL), and purified by GSH-sepharose affinity, ion exchange, and gel filtration chromatography in buffers 1 and 2, as described (Eckert & al., Roll-Mecak & Vale, 2008). All mutants were generated following the QuickChange protocol (Agilent Technologies, Santa Clara, CA, U.S.A.).

A codon-optimized version of full-length human katanin was synthesized commercially (MorphoSys/Sloning; Planegg, Germany). The synthetic gene was sub-cloned into pFastBac1 by PCR. In a second cloning step, EGFP with an N-terminal FLAG-binding sequence (DYKDDDDK) was introduced at the 5′ end of katanin's coding sequence by PCR. Recombinant baculovirus was prepared according to manufacturer's protocol (Invitrogen). The bacmid was isolated from *E. coli* DH10Bac and used for transfection of Sf9 (*Spodoptera frugiperda*) cells (Invitrogen). The virus-containing supernatant was used to infect fresh Sf9 cells for 72 h. This procedure was repeated twice to amplify the baculovirus. For expression, Sf9 insect cells were grown in SFM-900 II media (Invitrogen) supplemented with 100 µg/ml gentamicin and 0.25 µg/ml fungizone (Invitrogen). GFP-katanin-p60 was expressed in 500 ml Erlenmeyer flasks (Schott-Duran) containing 250 ml of SFM media, complemented with 5% FBS serum (Invitrogen). The cells were harvested 72 h post infection by 1000 g centrifugation, resuspended in buffer 3 with protease inhibitor cocktail (Roche Diagnostics), and lysed with a French pressure-cell (Fisher Scientific). Cell debris was removed by centrifugation (40.000 g for 30 min at 4°C). The protein was purified by affinity to anti-FLAG M2 affinity gel (Invitrogen). The cell lysate was incubated with the slurry and agitated for 60 min at 4°C, washed three times with buffer 3 and eluted with buffer 4. Finally, the protein was concentrated over a spin column (Millipore). Katanin concentrations were determined by extinction at 280 nm in a NanoDrop ND-1000 (Peqlab) spectrophotometer.

The N-terminal katanin domains (Kat1, encoding amino acids 1–55, Kat2, amino acids 56–181, and Kat3, amino acids 182–491), and the coiled coil spastin dimer were also cloned as FLAG-tagged proteins, and expressed and purified as the full-length katanin construct.

*Microtubules* were isolated from pig brain as described [13], [14]. Porcine brains used in this study were obtained from the Bayerische Landesanstalt für Landwirtschaft, Institute of Animal Breeding (Poing, Germany). The neuronal tissue was handled according to bio-safety rules and good laboratory practice. Microtubule concentrations were determined photometrically at 280 nm in the presence of 6 M guanidinium hydrochloride, and thus always refer to the concentration of tubulin dimers in polymerized microtubules [15]. Fluorescent microtubules were prepared as described [16], [17].

### Limited proteolysis with subtilisin

The following protocol was based on [18]. Tubulin (50 µl of 3 mg/ml) and Alexa Fluor 555-tubulin (1 µl, ∼1 mg/ml) were co-polymerized in BRB80 in the presence of 1 mM GTP at 36°C for 30 min. To stabilize the microtubules 20 µM paclitaxel (Invitrogen) was added, followed by incubation at 36°C for another 30 min. Proteolysis was accomplished by incubation with subtilisin A (Sigma; tubulin:subtilisin = 1∶0.8) at 36°C for 45 min in BRB80 with paclitaxel. The reaction was stopped by the addition of 4 mM PMSF in isopropanol (Fluka). After incubation at room temperature for 15 min, microtubules were sedimented at 40.000 g for 30 min. Pellets were washed with and resuspended in BRB80 with 20 µM paclitaxel and 1 mM GTP. The result was checked by SDS-PAGE.

### Hybrid microtubules

We formed Alexa Fluor 555-labeled, stabilized, and subtilisin-treated microtubule seeds as described [19]. Native, unlabeled tubulin was then added to elongate the microtubule seeds in the presence of 20 µM paclitaxel. As a result, only the subtilisin-treated microtubule parts were fluorescent. These hybrid microtubules were then mixed in reaction vials with GFP-spastin or GFP-katanin at different ratios. The binding was determined by fluorescence microscopy.

### Microtubule co-sedimentation assay

To quantify the binding behavior of spastin to microtubules in solution, co-sedimentation assays were used [20], [21]. Unless otherwise stated, a fixed concentration of microtubules was incubated with increasing concentrations of spastin in BRB80 buffer at room temperature. The assay volume was 80 µl. Nucleotides were added as desired. After the addition of all components (typically between 1 and 5 minutes), the reaction mixture was centrifuged at room temperature at 100,000 g for 10 minutes. A volume of 50 µl was removed and stored for analysis. The remaining supernatant was discarded; the sediment was washed with BRB80 buffer, and dissolved in SDS sample buffer in the original volume (80 µl). The sediment fractions were compared to the supernatant fractions on a SDS-gel. The amounts of spastin in pellet and supernatant were quantified by staining with Coomassie Blue, photography by a CCD camera (Peqlab), and the “Gel Loading Macros” in ImageJ.

The stoichiometry spastin: (tubulin dimer) was determined by plotting the concentration-dependent binding curves (x-axis: spastin concentrations in the supernatant fractions, y-axis: concentrations in the pellet fractions). The saturation curves extrapolates to the maximum concentration of bound spastin and thus allows estimating the binding stoichiometry [21].

### Electron microscopy

Electron microscopy was performed using carbon grids of the type FCF400-Cu square mesh (Electron Microscope Sciences, Hatfield, Pennsylvania). Grids were first plasma cleaned. Small volumes of spastin or katanin in buffer 2, or microtubules and mixtures of mutant spastin or katanin and microtubules in buffer BRB80/paclitaxel were then pipetted on the carbon grids and incubated for 5 seconds. The surfaces were then washed twice with water and fixed in uranyl formate [22]. After air-drying, images were taken using a Philips CM 100 transmission electron microscope equipped with a 4 megapixel camera (AMT, Woburn, Massachusetts). For particle averaging, the software EMAN2 was used [23], http://blake.bcm.edu/emanwiki/EMAN2). Particles were selected manually; classes were built on an average of approximately 20 particles per class.

### Activity assays

The severing activity of GFP-spastin was assessed in microscopic assays [8]. Flow cells were prepared and coated with anti-tubulin antibodies (0.2% monoclonal anti-TUB 2.1; Sigma-Aldrich Corp.St. Louis, MO, U.S.A., in BRB80). Then, the solution was exchanged against 5% pluronic-F127 (Sigma-Aldrich Corp.St. Louis, MO, U.S.A.) in BRB80. After 5 min, microtubules in BRB80 containing 20 µM paclitaxel (LifeTechnologies Invitrogen) were attached to the antibody-coated surface. The quality of the flow chamber was checked in the microscope before the chamber was excessively washed with BRB80/paclitaxel. The assay was started by addition of spastin and 1 mM ATP. Severing was recorded by a Hamamatsu C-9100 front-illuminated CCD camera, and analyzed by the Olympus CellR software (Olympus Europa, Hamburg, Germany). The analysis of severing rates was done in ImageJ [24] and IGOR Pro (Wavemetrics).

The enzymatic ATPase activity for the coiled-coil Δ227 spastin construct was measured as described [25]. Briefly, ATP consumption was coupled to NADH oxidation by phosphoenol pyruvate, lactate dehydrogenase and pyruvate kinase, and followed in a spectrophotometer at 340 nm. The turnover numbers were calculated per subunit.

The purity of ATP-γS (Adenosine-5-(γ-thio)-triphosphate; Jena Biosciences, Jena, Germany) was checked by HPLC [8]. 20 µl of a 1 mM nucleotide solution was applied to a reversed phase C18 column (Gemini-NX, 3 µm, 100A, 100×4.60 mm) and eluted with 10 mM tetrabutyl ammoniumchloride, 10 mM K2HPO4, 25% acetonitrile, pH 7.0, at a flowrate of 1 ml/min.

### Microscopic assays

Flow cells were prepared and coated as described for severing assays [8]. The assay was started by addition of 5–10 nM GFP-spastin/GFP-katanin and 1 mM of nucleotide. For experiments in which different ionic strength-conditions were applied to the flow cell, the required volume of NaCl (1 M) was added to buffer 5 yielding a final assay concentration of 80, 160 or 300 mM NaCl.

Single molecule images were acquired with a Hamamatsu C-9100 front-illuminated CCD camera. Total internal reflection fluorescence (TIRF) microscopy used a home-built laser system around an Olympus IX71 microscope and a high numerical aperture objective (100×, NA = 1.45). TIRF excitation was achieved using a solid-state laser to visualize GFP. MTs were imaged via 535-nm laser. An oxygen scavenging system of glucose oxidase and catalase was employed to reduce photobleaching and photodamage during illumination with the laser [26]. The standard exposure time was 200 ms, the frame interval was 0.229, 1 and 10 s. To measure binding dwell times and diffusion, we imaged with 100 ms exposure without delay to capture short time binding events. Control experiments without GFP-spastin/katanin or with inactive mutants E442Q/E309Q were performed to make sure we did not damage MTs during regular imaging.

### Data analysis

The data were recorded as 16-bit images using CellR software (Olympus Biosystems). Microscope data were exported as 16-bit tiff stacks. The motion of single GFP-spastin/katanin molecules was visualized by kymographs generated with the Multiple Kymograph plugin for ImageJ (J. Rietdorf, FMI Basel and A. Seitz; EMBL, Heidelberg). The quantitative analysis was done in two steps: First, the microscope image sequences were imported into the program OpenBox [27]. This program has a routine that localizes selected spots by 2-dimensional Gaussian fits. The time sequence of x-y pairs generated by OpenBox were exported and analyzed by a self-written function in IGOR Pro (Wavemetrics). This function calculates the distances of each spot from frame to frame, and tabulates the absolute (oriented) displacements, and the mean squared displacements (m.s.d.). Histograms of absolute displacements were used to determine an orientational preference. Plots of the m.s.d. against the time show local variations that were exponentially distributed. The calculation of the diffusion coefficient was based on the characteristics of the distribution (Supporting Figure S3).

The frequency of severing was obtained by counting the number of breaks in every microtubule by hand [8], [12]. The number of severing events in a single movie was divided by the total length of the microtubule at the start of the movie, and divided by the time from assay start to severing. The frequency of binding was determined by counting the number of events manually. The duration of association was measured from kymographs.

## Results

### Microtubule Interaction and Diffusion

Both spastin and katanin are microtubule-severing enzymes, and separate microtubules internally into pieces after a phase of incubation [8], [10], [28]. They contain highly similar AAA domains, which are responsible for ATP turnover and hexamer formation (Figure 1). These similarities suggest a similar microtubule-severing mechanism [2]. However, microtubule binding of spastin and katanin seems to be accomplished by non-homologous parts N-terminal to the core catalytic domains [5], [6], posing the question how microtubule binding and severing activities are connected.

**Figure 2:**
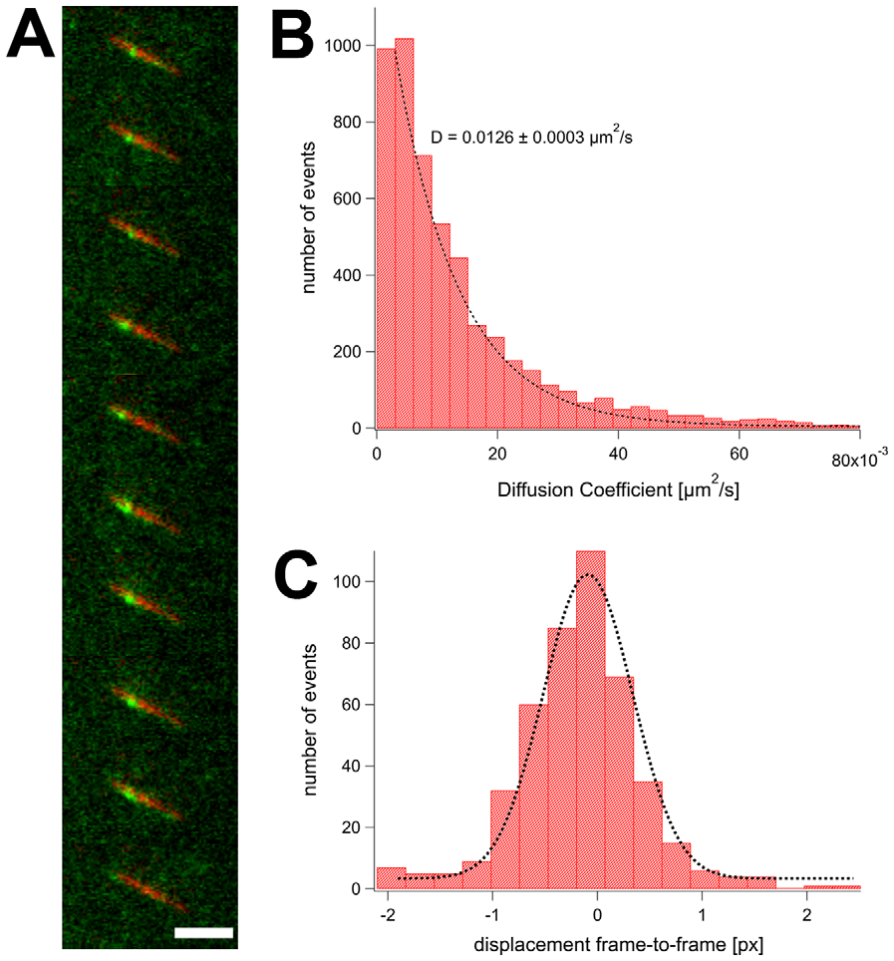
Diffusion of Spastin along Microtubules. Panel A shows an image sequence of GFP-Δ227 HsSpastin (green) on a microtubule (red). The scale bar is 1 µm, the time between two frames was 1 s (for analysis, the full time resolution was used; see Supporting Movie S1). Panel B shows the distribution of diffusion coefficients of 30 molecules. Each film sequence was analyzed frame by frame, and the x-y position of the molecule of interest was localized in each frame (see Material and Methods). The mean-squared displacements were calculated and plotted against time. These traces were used to calculate the 1-dimensional diffusion coefficient over a sliding window (size = 5 frames). The distribution of all local diffusion coefficients of all molecules is plotted in the histogram. Panel C plots a histogram of distances from frame to frame with a Gaussian fit.

The interaction between katanin and microtubules has been investigated by domain mapping and biophysical studies [5]. It turned out that microtubule-bound katanin moves diffusively along microtubules before it severs the filament. To find out whether spastin shows a similar behavior, we observed GFP-Δ227 HsSpastin in TIRF microscopy assays (Figure 2; Supporting Movie S1). This construct comprised amino acid residues 227 to 616 of the human spastin protein, fused to a N-terminal EGFP fluorescence tag (Material and Methods). The Δ227 HsSpastin construct has been characterized previously, and it has been found that it shows severing and ATPase activities with properties very similar to full-length spastin [6], [8]. The N-terminal GFP fusion construct used here was able to sever microtubules in vitro at spastin concentrations similar to that of the GFP-free variant, and consumed ATP at the same maximal turnover rate as its parent construct (3.2 to 3.5 s^-1^ (Supporting Figure S1) and [8]). The severing activity of native and GFP-tagged spastin occurred only above a certain threshold value of approximately 150 to 250 nM enzyme (Supporting Figure S2 and Supporting Movie S3; [8]). At protein concentrations between 5 and 10 nM GFP-Δ227 HsSpastin, no severing occurred, but we observed single fluorescent spots moving along microtubules. In the presence of 1 mM ATP, most of the spots moved along the filaments. To locate the positions accurately, the fluorescence signals were fitted by a two-dimensional Gauss function, and tracked during their movements (Figure 2B and Supporting Figure S3). It turned out that the particles moved without detectable directional bias, and that their m.s.d. were linear with time, which is characteristic for diffusional mobility. To determine the diffusion coefficient, we analyzed the m.s.d. traces, and fitted them locally, using a sliding window that averaged the slope over a variable number of frames (Material and Methods; Supporting Figure S3). The local diffusion coefficients were distributed exponentially, indicative for a random process and excluding saltatory events (Figure 2). The apparent one-dimenstional diffusion coefficient was 0.0126±0.0003 µm^2^/s (average and fitting error), regardless whether the average was calculated as the arithmetic mean or the decay of the exponential function.

**Figure 3:**
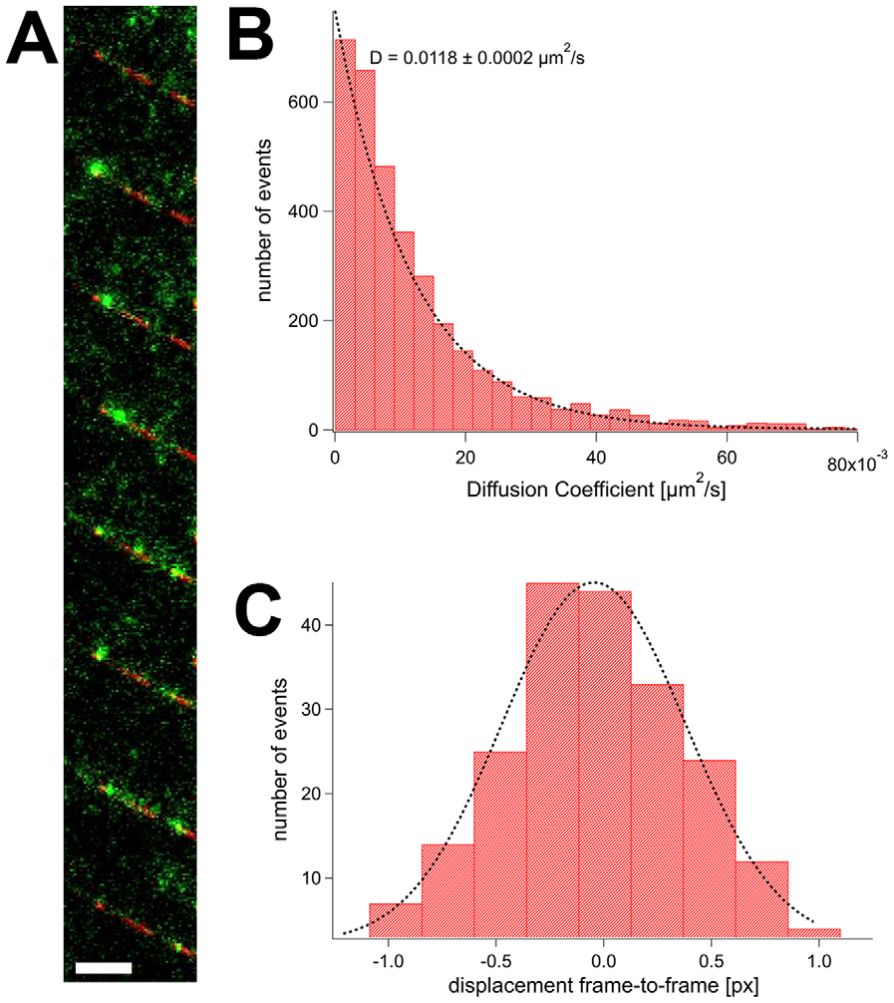
Diffusion of Katanin along Microtubules. Panel A shows an image sequence of GFP-FLAG HsKatanin (green) on a microtubule (red). The scale bar is 5 µm, the time between two frames was approximately 1 s (for analysis, the full time resolution was used; see Supporting Movie S2). Panel B shows the distribution of diffusion coefficients of 30 molecules, analyzed as in Figure 2. The distribution of all local diffusion coefficients of all molecules is plotted in the histogram. Panel C plots a histogram of distances from frame to frame with a Gaussian fit.

We determined the diffusion coefficient of GFP-katanin using the same setup (Figure 3 and Supporting Movie S2). In this case, we used the full-length construct with an N-terminal EGFP tag. In the presence of ATP, GFP-katanin showed an average diffusion coefficient of 0.0118±0.0002 µm^2^/s (average and fitting error), very similar to GFP-spastin. Diaz-Valencia et al. published a lower value (0.0033 µm^2^/s) but considered a large population of immotile particles for their calculation [12]. Noteworthy, the diffusion of spastin and katanin was sensitive to the kind of nucleotide present. In the presence of 1 mM ATP-γS the diffusion was less pronounced, and in the presence of 1 mM AMPPNP it was almost absent (Supporting Figure S4 and S5). Also, fluorescent spots were brighter under AMPPNP than under ATP conditions (Supporting Figures S4 and S5). However, we were unable to quantify the extent reliably, and to decide whether spastin hexamers show a specific form of diffusion. We tried to count the number of bleaching steps of fluorescent spots, and, in fact, often observed successively lower plateaus of fluorescence intensity. However, the outcome of the analysis was strongly dependent on the selection of particles, preventing unbiased conclusions.

### Microtubule Landing and Dissociation Rates

Our TIRF movies contained more information than only the diffusion behavior. Using kymographs, we were able to count landing rates of fluorescent spots (Table 1 and 2). In the presence of 10 nM enzyme, the landing rate per 10 µm of microtubule and minute was 24.2 ((10 µm)^−1^ min^−1^ (10 nM)^−1^ spastin), and 15.8 ((10 µm)^−1^ min^−1^ (10 nM)^−1^ katanin) in buffer 5. The landing rates were measured in the presence of ATP, ATP-γS and AMPPNP (Table 1). For spastin, ATP and ATP-γS were indistinguishable and led to landing rates of approximately 22 per 10 µm microtubule length and 60 s. The rate was approximately half as large for AMPPNP. The landing rate was highly salt-dependent, and decreased to 1.6 ((10 µm)^−1^ min^−1^ (10 nM)^−1^ spastin) and 1.9 (10 µm^−1^ min^−1^ (10 nM)^−1^ katanin) at an ionic strength of 365 mM and 1 mM ATP (Table 2).

**Table 1:** Nucleotide dependence of microtubule interaction.

	Nucleotide	dwell time	landing rate	k_off_/k_on_
units		[s]	[(10 µm)^−1^ · (60 s)^−1^ · (10 nM)^−1^ katanin]	[10 nM 10 µm]
Spastin	ATP	10.07	22.72	0.262
	ATP-γS	55.68	21.24	0.051
	AMPPNP	>253.69	13.39	0.018
Katanin	ATP	4.02	14.08	1.060
	ATP-γS	10.05	8.73	0.684
	AMPPNP	>245.9	2.06	0.118

**Table 2:** Salt dependence of microtubule interaction.

	NaCl	Ionic Strength	dwell time ± s.e.m.	landing rate	k_off_/k_on_
units	[mM]	[M]	[s]	[(10 µm)^−1^ · (60 s)^−1^· (10 nM)^−1^ katanin]	[10 nM 10 µm]
Spastin	80	0.145	not applicable	24.21	0.397
	160	0.225	6.4±0.8	4.07	3.219
	300	0.365	4.2±0.1	1.61	16.274
Katanin	80	0.145	not applicable	15.77	0.609
	160	0.225	5.6±0.2	4.64	2.823
	300	0.365	2.5±0.0	1.93	13.576

**Figure 4:**
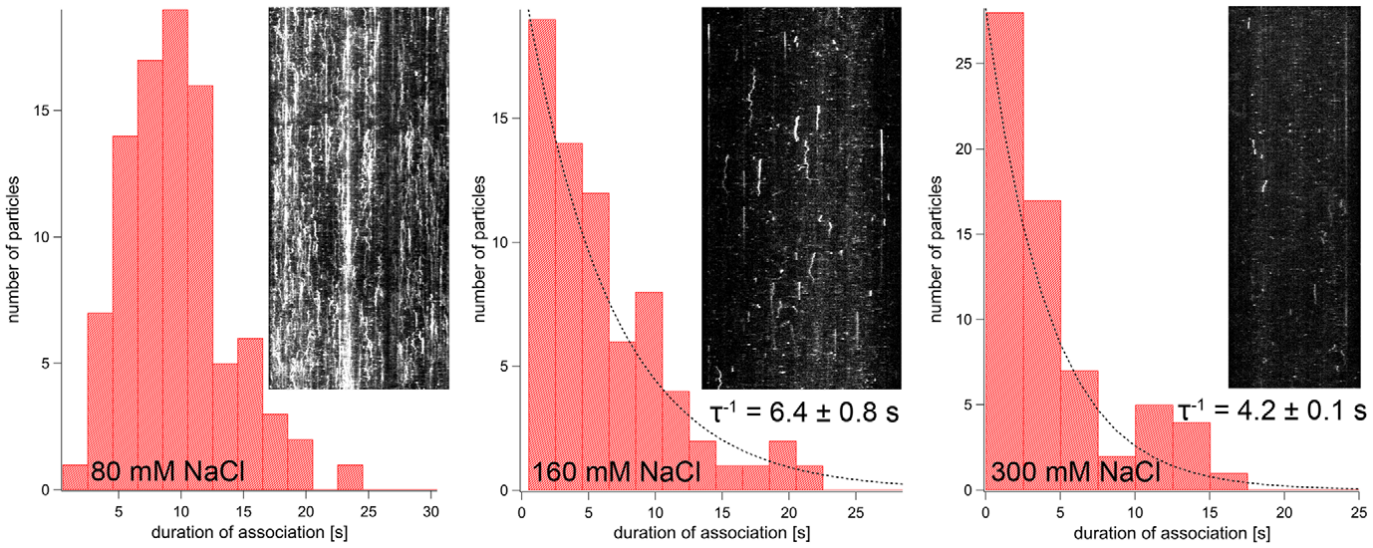
Spastin Dissociation Rates. The figure shows dwell-time distributions of GFP Δ227 HsSpastin on microtubules at different ionic strengths. The histograms contain insets showing example kymographs of binding events. The dotted lines are mono-exponential curve fits that were used to calculate the lifetime, τ.

**Figure 5:**
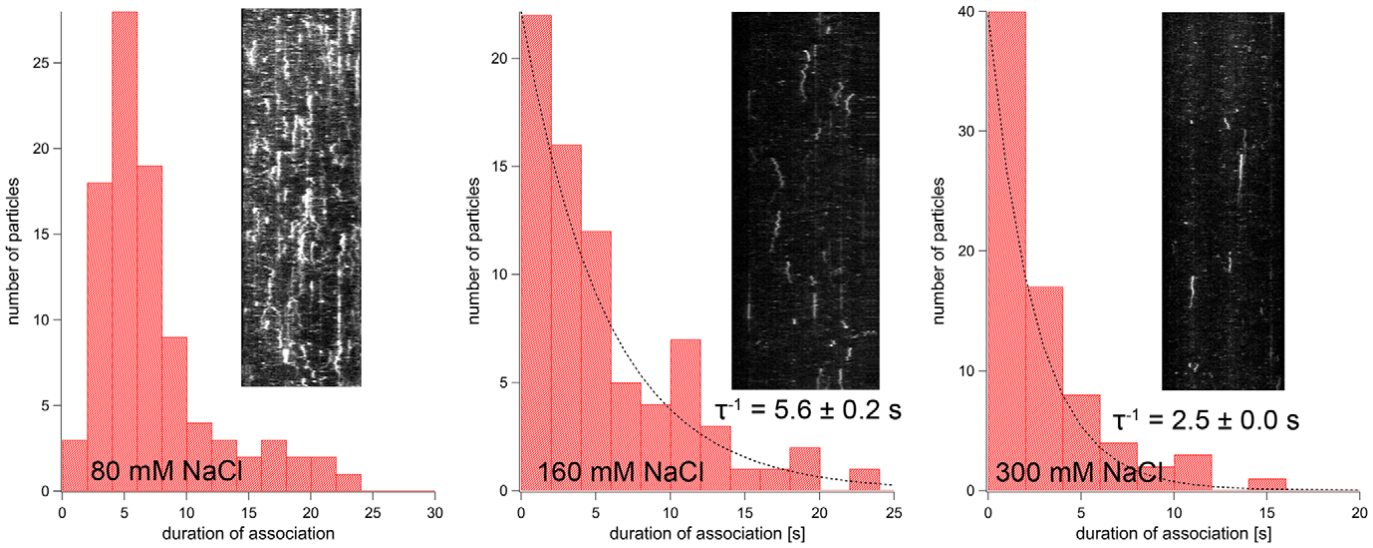
Katanin Dissociation Rates. The figure shows dwell-time distributions of GFP-HsKatanin on microtubules at different ionic strengths. The histograms contain insets showing example kymographs of binding events. The dotted lines are mono-exponential curve fits that were used to calculate the lifetime, τ.

The dwell times of fluorescent spots at microtubules were also determined from kymographs. AMPPNP almost completely prevented the dissociation of spastin and katanin. Once bound, the fluorescent spots mostly remained bound until the end of the movie clip. Therefore the values given in Table 1 are lower limits of the real dwell time. We observed the shortest dwell times under ATP conditions (10 s for spastin, 4 s for katanin). ATP-γS increased these times 5–6 fold (spastin) or 2.5-fold (katanin). The dwell times of both enzymes also showed a clear dependence on the ionic strength (Figures 4 and 5; Table 2). They ranged between 9.6±4.1 s (average and standard deviation for spastin, 145 mM ionic strength) and 4.2±0.1 s (spastin, 365 mM ionic strength, exponential lifetime ± s.e.m.), and 7.3±4.7 s (katanin, 145 mM ionic strength) and 2.5±0.0 s (katanin, 365 mM ionic strength, exponential lifetime ± s.e.m.). The dwell times in the presence of 160 and 300 mM NaCl were distributed mono-exponentially, and the lifetimes given in table 2 lay close to the inverse exponential decay constants. At 80 mM NaCl the dwell times were not distributed exponentially and showed increasing numbers up to 4 to 8 seconds, possibly because of multiple or unspecific ionic interaction sites. Due to the lack of a testable model, we were unable to use curve fitting for the determination of dwell times, and used the mean value.

In summary, both dissociation rate and landing rate are salt-dependent, but the former ones are less sensitive towards salt than the binding rates. Together, these results show that spastin as well as katanin bind to microtubules via ionic interactions. To follow up this issue further, we looked for charged residues in microtubules and spastin that might be involved in binding.

### Subtilisin-treated Microtubules

**Figure 6:**
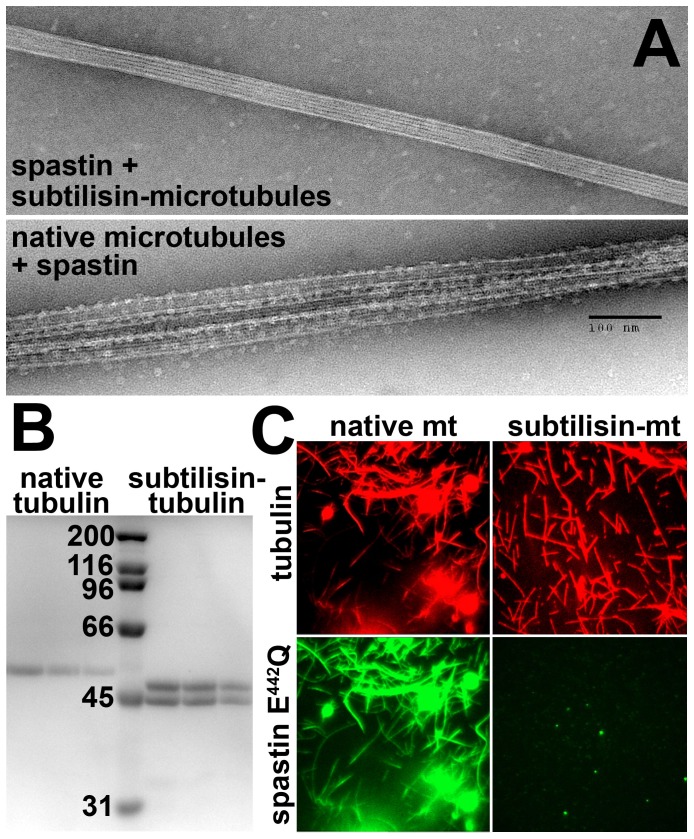
Spastin Interaction with Subtilisin-treated Microtubules. Panel A shows negative stain electron micrographs of microtubules and the spastin E442Q mutant in the presence of 1 mM ATP. In the top part, subtilisin-treated microtubules were used, the lower part shows native microtubules. Binding and bundling occurs only with native microtubules. Panel B shows a SDS-gel of tubulin before and after subtilisin-proteolysis. Panel C shows TIRF microscopy images of Alexa Fluor 555 microtubules (red) and GFP Δ227 HsSpastin E442Q (green). The GFP spastin construct decorates and bundles only untreated microtubules.

On the microtubule side, the charged C-terminal end (‘E-hook’ [29]) is an obvious candidate for ionic interactions. It has been removed by limited proteolysis with subtilisin (Figure 6B, [30]). Using this method, it has been proposed that severing of both spastin and katanin depends on the presence of the E-hook [5], [31]. Whether the lack of severing is due to a lack of binding, however, has not been investigated. We therefore performed microscopic and biochemical assays on untreated and subtilisin-treated microtubules (Figure 6). For most of these assays, we used inactive Walker B mutants that are unable to disassemble microtubules. Hence, the spastin mutant E442Q was used for binding assays [6], [7]. Curiously, this mutant showed a strong bundling effect when incubated with microtubules in the presence of ATP (Fig. 6C). The bundling was strictly dependent on the presence of the native E-hook and disappeared when subtilisin-treated microtubules were used. Electron micrographs gave the same picture (Figure 6A). In addition, a decoration of microtubules with ring- and dot-shaped structures occurred on native microtubules, while subtilisin-treated microtubules were essentially devoid of any decoration. Most likely, native microtubules were decorated by hexameric spastin ring because the E442Q spastin mutant forms hexamers in the presence of ATP [7], [8], [28].

**Figure 7:**
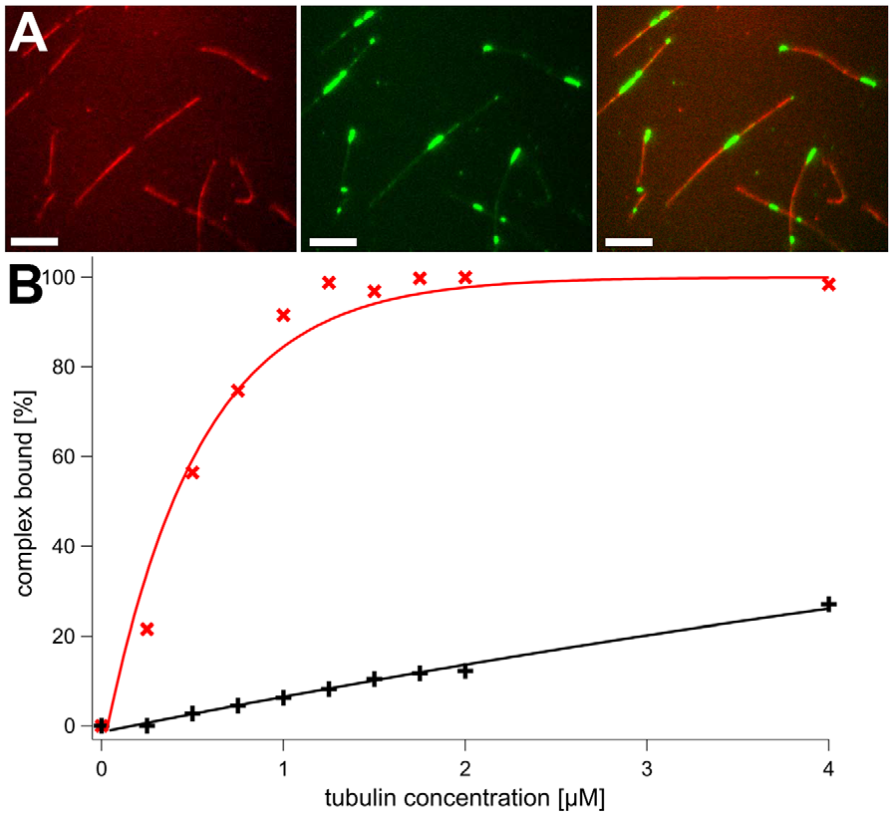
Katanin Interaction with Subtilisin-treated Microtubules. Panel A shows fluorescence microscopy images of GFP HsKatanin (green) and microtubules. The fluorescent, red parts of microtubules are subtilisin-treated, native stretches are unlabeled. Panel B shows a comparison of binding curves obtained by co-sedimentation of HsKatanin and microtubules. The fraction of bound enzyme was determined by SDS-PAGE of supernatants and pellets. The lines are connecting the data points without a specific model.

The analogous Walker B katanin mutant (E309Q) did not bundle microtubules. To characterize its microtubule-binding properties, we used hybrid microtubules nucleated from subtilisin-treated seeds. These seeds were produced in the presence of Alexa Fluor 555-labeled tubulin, and purified over a sucrose cushion to remove subtilisin and other impurities. Afterwards, non-fluorescent tubulin was used to elongate the seeds, and thus to produce hybrid microtubules with strongly fluorescent parts lacking the E-hook, and dim parts with native C-termini. GFP-katanin clearly localized only at dim, native parts of the filament when incubated at a 1∶32 stoichiometric ratio (Figure 7). Interestingly, at very high ratios (1∶2 katanin/tubulin), the bright, subtilisin-treated parts were also decorated with katanin. We tested this observation in cosedimentation assays. The assay was performed at a fixed E309Q-katanin concentration and variable microtubule concentrations (Figure 7B). For native microtubules, we obtained a hyperbolic saturation curve that extrapolated to 100% of the katanin used. The half-maximal saturation was reached at approximately 0.4 µM microtubules. Subtilisin-treated microtubules showed a much lower affinity that did not approach saturation even under the highest microtubule concentration used. At 4 µM microtubules, less than one third of the katanin was bound, suggesting an affinity that is much more than 10-fold reduced. The digested microtubules might contain a certain amount of E-hook, although the SDS-gel suggested an invisible amount of contamination (Figure 6B). Therefore, katanin seems to have a certain affinity for E-hook depleted microtubules.

### Microtubule Binding Site of Human Spastin

As the counterpart of the negatively charged E-hook of microtubules we searched for basic residues in spastin. Domain mapping studies have revealed that spastin comprises several functional parts [6]. In human spastin, the microtubule binding function has been allocated to the region between residues 270 and 328 (MTBD, Figure 1). A construct lacking this part was unable to bind to microtubules [6]. In agreement, in our hands a Δ323 spastin construct failed to bind and sever microtubules. The MTBD region is predicted to form no ordered secondary structures but is highly charged (18 basic and 4 acidic amino acid residues, as well as 4 histidine residues in human spastin). Most obvious is a cluster of four positively charged residues (R^309^KKK), which we mutated to R^309^QQQ. Curiously, in addition to parts of the linker, *Drosophila* spastin required determinants from the MIT domain to bind to microtubules [7]. In agreement, in *Drosophila* spastin, the large positive net charge of the linker is not conserved. This observation raises the questions how specificity is achieved for human spastin, and how microtubule binding is coupled to the severing process.

**Figure 8:**
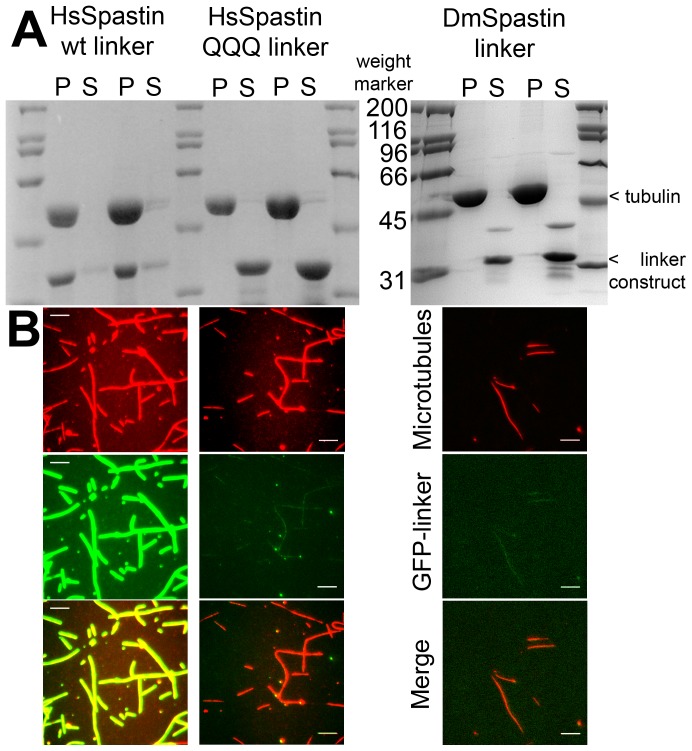
Microtubule Binding Properties of the Spastin Linker (MTBD) Domain. Panel A shows the results of a co-sedimentation assay of microtubules and GFP-linker domain constructs. From left to right: GFP-HsSpastin wild type linker domain (approximately 34 kDa) was mixed with microtubules (50 kDa) at a stoichiometric ratio of 1∶1. The microtubule-bound fraction appears in the pellet (P) of ultracentrifugation, the unbound fraction in the supernatant (S). Two amounts were analyzed by SDS-PAGE. Lanes 7–10 show the same experiment with the triple K^310^KK>Q^310^QQ mutation. The right SDS-gel shows a co-sedimentation assay with the analogous region from *D. melanogaster* spastin. Weight markers (lanes 1, 6, 11 left gel, and lanes 1 and 6 right gel) are given in kDa. Panel B: Binding of the GFP-linker constructs to microtubules in a TIRF microscopy assay.The top row shows Alexa Fluor 555 microtubules in red, the middle row GFP-linker constructs in green, the bottom row an overlay of red and green channels. All images were taken at the same gain and exposure time. The images show that the GFP HsSpastin wild type linker (left column) coats all microtubules densely, while the mutant (center column) and the *Drosophila* (right column) constructs fail to bind to a significant degree.

To characterize the microtubule binding properties, we performed co-sedimentation and microscopic assays with the isolated MTBD domain, as identified by White et al., tagged with GFP [6]. To this end, we incubated each of the GFP-MTBD constructs with microtubules at a stoichiometric ratio of 1∶2 (GFP-MTBD/tubulin) in BRB80 buffer. The microtubule-bound fraction was separated from the unbound fraction by ultracentrifugation. The human wild type GFP-MTBD construct was found almost completely in the pellet fraction, showing that it was bound to microtubules (Figure 8). In contrast, all of the Q^310^QQ-mutant, and all of the DmLinker construct remained in the supernatant, demonstrating that the binding affinity is based on the triple lysine motif. Microscopic analyses gave the same result. While all microtubules were fully decorated with GFP-MTBD protein, only traces of triple Q mutant bound to microtubules (Figure 8B).

**Figure 9:**
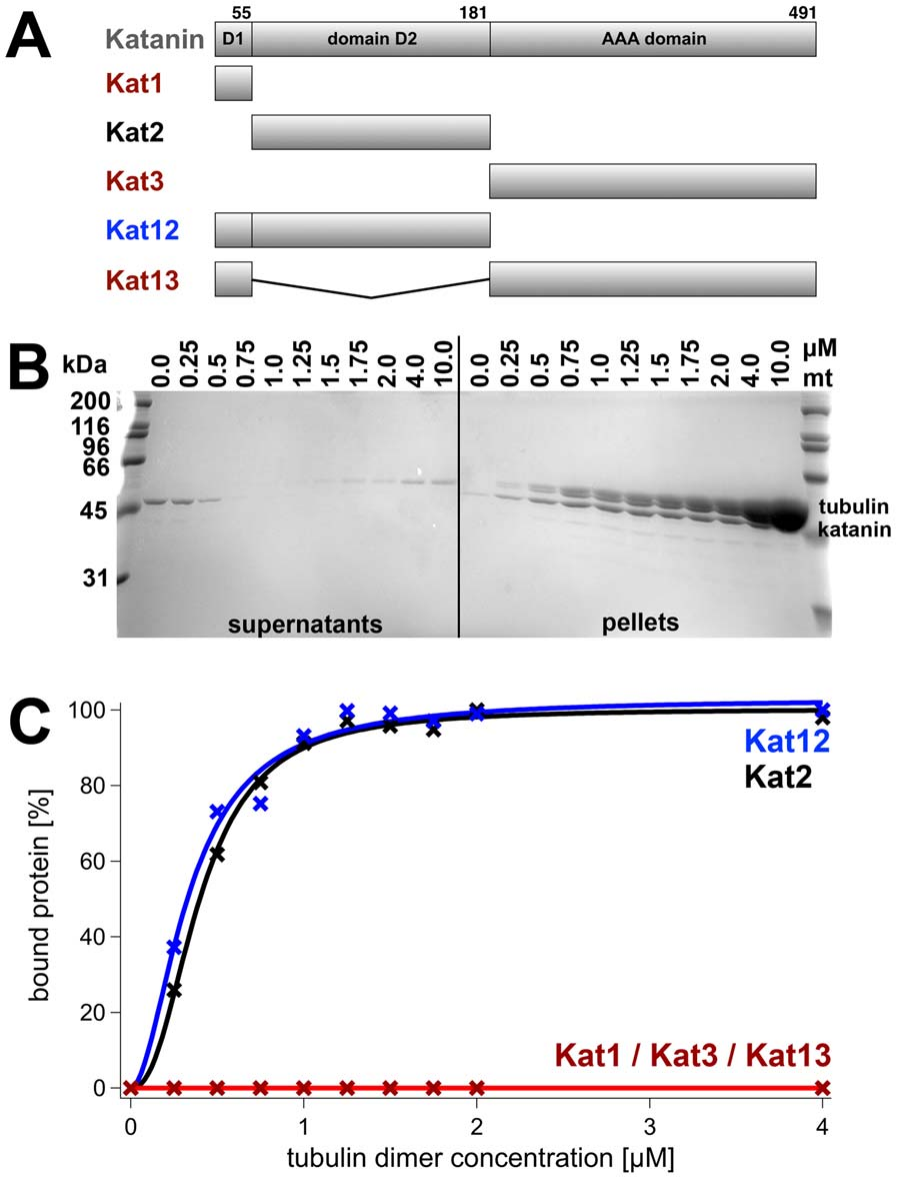
Domain-Mapping of the Katanin-Microtubule Interaction. Panel A displays the constructs used for katanin binding experiments. Panel B shows a quantitative SDS-gel of supernatants (unbound) and pellets (microtubule-bound) of an in vitro binding assay of truncated katanin constructs and microtubules. Increasing microtubule concentrations (0 to 10 µM) were incubated with a fixed katanin construct concentration (1 µM). The density of the katanin construct band was plotted against the microtubule concentration and fitted to a Hill function (panel C). The half-maximal saturation was reached at 0.34 µM (Kat12) and 0.40 µM (Kat2). Only constructs containing domain 2 were able to bind to microtubules.

For comparison, we also tested katanin's microtubule-binding properties. In previous publications, the importance of the N-terminal, non-AAA domains has been found [11], [32]. We generated five truncation constructs with boundaries analogous to [32] (Figure 9A): either domain 1 alone (Kat1), or domain 1 and 2 (Kat12), or domain 1 and 3 (Kat13), or domain 2 alone (Kat2) or domain 3 (Kat3). Constructs comprising all domains, or domains 2 and 3 were active in severing assays, and therefore not suited for binding assays. Co-sedimentation assays showed that the constructs Kat2 and Kat12 bound to 100% to microtubules with the same affinity of approximately 0.3 µM half-saturation (Figure 9B and C). None of the other constructs was found in the pellet fraction, showing that domain 2 is necessary and sufficient for microtubule binding.

### Nucleotide Dependence of Microtubule Binding

**Figure 10:**
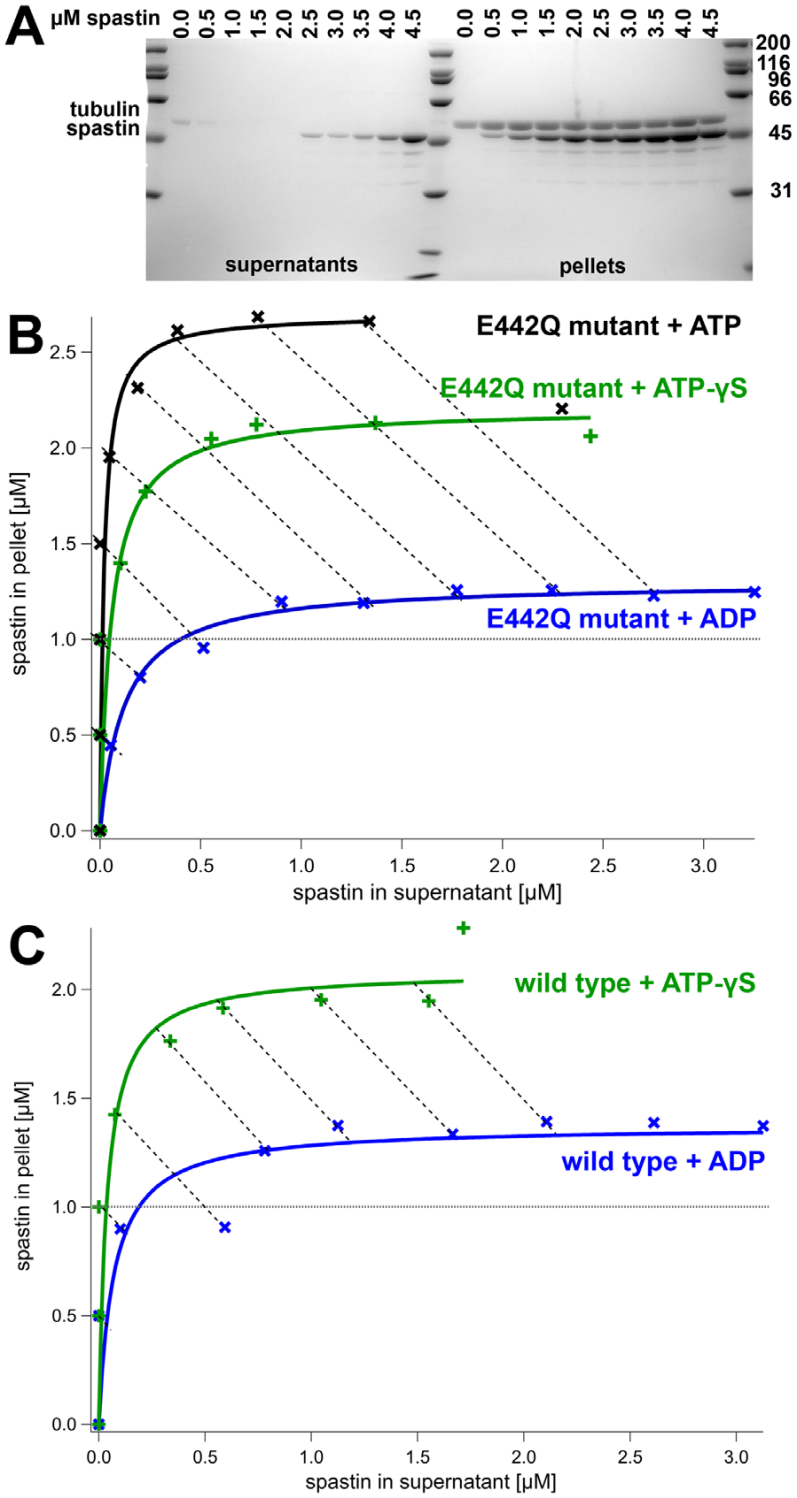
Nucleotide-Dependence of the Spastin-Microtubule Interaction. Panel A shows a SDS-gel of co-sedimentation assays with spastin (E442Q mutant, 1 mM ATP) and a constant concentration of microtubules (1 µM; indicated by a dotted line). With increasing spastin concentrations, an increasing amount of protein is co-sedimented with microtubules. Panel B: Densitometric analysis allowed plotting of the spastin concentrations bound to microtubules against free spastin concentrations. The horizontal dotted line indicates the concentration of tubulin used in the assays. The diagonal dashed lines connect points with the same total concentration of spastin. The concentrations increase towards the upper right. Steeper curves reflect a higher affinity. Panel C shows the same experiment for wild type spastin.

**Figure 11:**
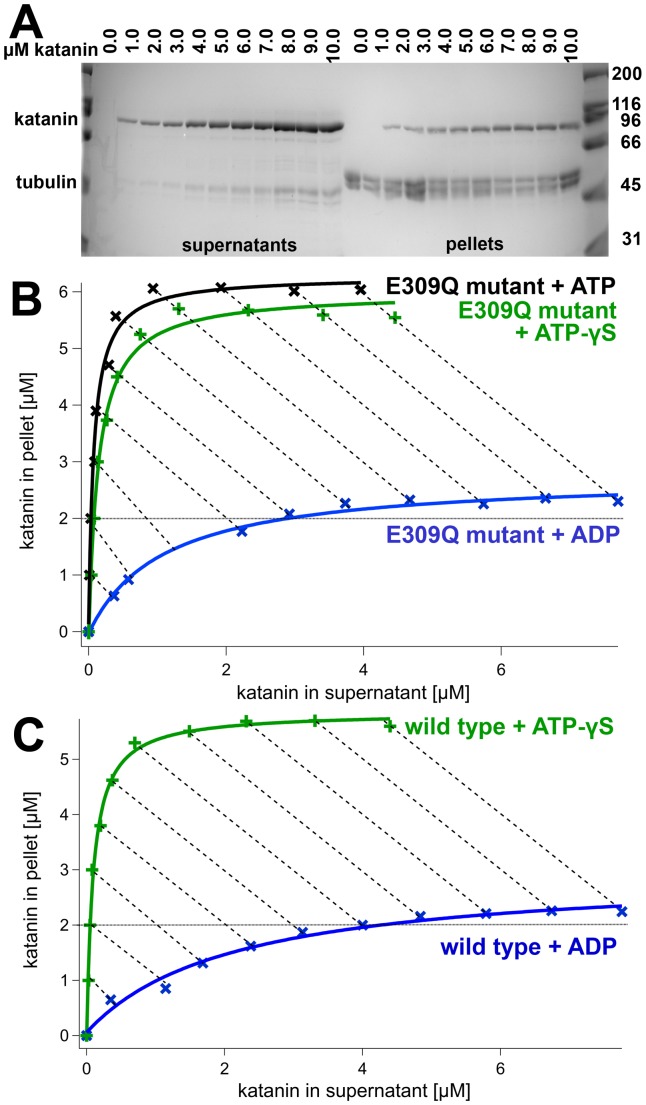
Nucleotide-Dependence of the Katanin-Microtubule Interaction. Panel A shows a SDS-gel of co-sedimentation assays with katanin (E309Q mutant, 1 mM ATP) and a constant concentration of microtubules (2 µM; indicated by a dotted line). With increasing katanin concentrations, an increasing amount of protein is co-sedimented with microtubules. Panel B: Plot of the densitometric analysis as in Figure 10. Panel C shows the same experiment for wild type katanin.

Motor proteins show a strongly nucleotide-dependent microtubule affinity [33]–[35]. To test whether this is the case for severing enzymes, too, we performed co-sedimentation assays in the presence of different nucleotides and nucleotide analogs (Table 3). The assay was set up at a constant microtubule concentration and variable enzyme concentrations [21]. This setup allows plotting the microtubule-bound fraction against the free fraction (Figures 10 and 11). The binding curves extrapolate to a maximal value of enzyme bound to microtubules, which reflects the binding stoichiometry enzyme:tubulin. In figures 10 and 11, diagonals are plotted connecting points with equal total enzyme concentration. Points lying further to the upper left along these diagonals show conditions with higher affinities. Using these criteria, spastin and katanin show the weakest microtubule affinity under ADP conditions. Katanin wild type is more sensitive towards the presence of either ADP or ATP-γS than spastin. The use of the Walker B mutants allows the use of ATP in these assays, and shows a similar behavior of katanin under ATP and ATP-γS conditions, whereas E442Q spastin's microtubule affinity is higher in the presence of ATP.

**Table 3:** Binding stoichiometry.

	Nucleotide [1 mM]	wild type enzyme∶tubulin	Walker B mutant enzyme∶tubulin
Spastin	ATP	n/d	2.7∶1
	ATP-γS	2.1∶1	2.2∶1
	ADP	1.4∶1	1.3∶1
Katanin	ATP	n/d	3.0∶1
	ATP-γS	2.8∶1	2.8∶1
	ADP	1.1∶1	1.1∶1

Surprisingly, the stoichiometry of binding was also affected by the nucleotide state. The ADP curve extrapolated to approximately 1.4 spastin subunits per tubulin dimer, the ATP-γS curve to 2.1. The E442Q mutant showed nucleotide-dependent binding stoichiometries, too, with values of 1.3 E442Q spastin per tubulin dimer under ADP conditions, or 2.7 (2.2) spastin monomers under ATP (ATP-γS) conditions. Katanin's binding stoichiometry extrapolated to 3.0 katanin subunits per 1 tubulin dimer under ATP (wild type, E309Q mutant) and 2.8 under ATP conditions (mutant E309Q), whereas ADP induced binding of approximately 1.1 bound subunits per tubulin dimer. This observation suggests that the microtubule-bound ATP state is of higher oligomeric order than the ADP state.

### Microtubule-stimulated ATPase Activity

**Figure 12:**
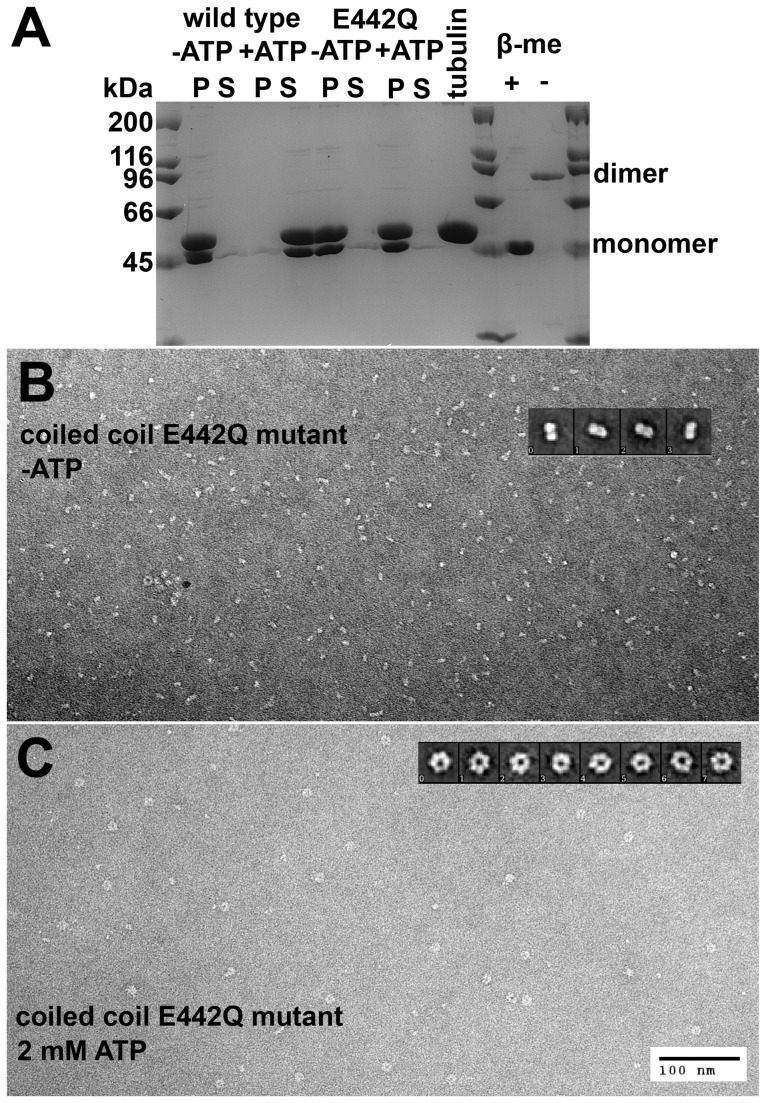
Electron Micrographs and SDS-PAGE of the Coiled Coil Spastin Construct. Panel A shows a SDS-gel with the analysis of an in vitro severing assay (left part) and an oligomerization assay. Lanes 1 and 11 are weight markers, lanes 2 to 9 results of the severing assay. The coiled coil spastin wild type construct was incubated with microtubules, and centrifuged after 15 min. Active spastin disassembles microtubules completely under these conditions, such that tubulin appears in the supernatant. Otherwise, microtubules are found in the pellet. Only the wild type coiled coil construct is active in the presence of ATP. The right part of the gel (lanes 12 and 13) shows the coiled coil wild type construct under reducing conditions (lane 12, 1 mM mercapto-ethanol) and oxidizing conditions (lane 13; omission of reducing agents). The coiled coil construct migrates as a dimer, due to a disulphide bridge introduced in the coiled coil domain. Panels B and C show electron micrographs of the E442Q mutant coiled coil construct without ATP, and with 2 mM ATP. Insets show particle averages of the predominant species. The averages were obtained with a semi-automated procedure implemented in EMAN2.

The fact that the stoichiometry of microtubule binding depended on the nucleotide suggested that oligomerization might occur. To support this notion, an artificial constitutive oligomer would be the most suited construct to test. Due to the difficulty of constructing and handling such a large constructs, we confined our studies to dimers. To force dimerization, we attached the *Drosophila* neck coiled coil domain to the N-terminus of Δ227 spastin. To facilitate coiled coil formation, we introduced a cysteine at the first coiled coil D position. It has been shown that this domain forms a stable two-stranded coiled coil [36]. In agreement, the coiled-coil-Δ227 HsSpastin construct was found to form dimers in electron micrographs and non-reducing SDS-PAGE (Figure 12).

**Figure 13:**
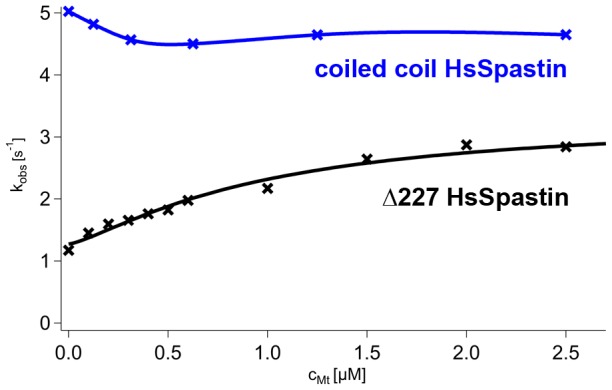
Microtubule-Dependence of the ATPase Activity of the Coiled Coil Spastin Construct. The figure shows the steady state ATPase turnover of wild type Δ227 HsSpastin (data from [8]) and the coiled coil construct. The activity of the reference construct is stimulated by microtubules according to a Hill curve (black line), the coiled coil construct lacks this feature. The blue curve is an interpolation of the data points and does not reflect a specific kinetic model.

To investigate the functional implications of the forced dimerization, we measured the microtubule-activated ATPase activity of this construct (Figure 13). The normal Δ227 HsSpastin construct shows a saturation curve with increasing microtubule concentrations that follows a sigmoidal dependence. In the absence of microtubules, the ATP turnover rate is ∼1 s^−1^, and increases to ∼4 s^−1^ at high microtubule concentrations. The coiled coil construct consumed ATP at an almost constant rate of ∼4 s^−1^ under all microtubule concentration. Even in the absence of microtubules, the turnover rate was as fast as under fully activated conditions, suggesting a correlation between microtubule binding and oligomeric state.

## Discussion

The microtubule-severing AAA ATPases spastin and katanin are believed to be active as homo-hexameric rings that may pull parts of the tubulin polypeptide chains through the central pore [6], [7]. Our data show that the human forms of spastin and katanin both bind to microtubules via unrelated non-AAA parts, and move along the filament diffusively with similar velocities. The diffusion coefficient of severing enzymes lies between those of dynactin (0.0015 µm^2^/s) and MCAK (0.36 µm^2^/s) [37], [38]. The diffusion of the isolated MTBD of human spastin was virtually indistinguishable from full-length spastin in the presence of ATP, indicating that it serves as the primary binding site. This observation, and the fact that the non-hydrolyzable nucleotide analogs ATP-γS and AMPPNP do not completely abolish diffusion, suggests that ATP hydrolysis is not required for diffusion. Instead, the diffusion seems to be powered by thermal energy, and its velocity largely dependent on the energy barrier between two E-hooks [39], [40]. This diffusion model explains the fact that the diffusion constants of spastin and katanin are similar, although the binding affinities turned out to be different. If the height and width of the potential barrier leading to dissociation were much larger than the barrier for lateral diffusion, a higher affinity would not be directly coupled to a slower diffusion.

In our work, we identified an essential RKKK-motif in spastin's MTBD, and found strong support for its interaction with the negatively charged ‘E-hook’ of tubulin. For katanin, we mapped the MTBD to region between residues 56 and 181, in agreement with previous publications [11], [32]. Iwaya et al. identified four residues in the vicinity of the start of our construct (R49, Q 53, K64 and K67), each of which was essential for an interaction with tubulin. Interestingly, the interaction was much stronger for unpolymerized tubulin. Our observation that microtubules are a strong binding partner agrees with experiments on *Arabidopsis* katanin [32]. However, Vantard and co-workers and our group used constructs that disrupt the tertiary structure of the N-terminal MIT domain, which was identified after we had started our work [11]. Still, the constructs used in these three studies contain common positively charged residues, and thus suggest that ionic interactions dominate the binding properties.

These observations show that the relation between microtubule binding and severing is very complex. For spastin, the non-AAA MTBD domain is necessary and sufficient for microtubule binding but cannot be responsible for severing [6]. On the other hand, the catalytically active AAA domain alone is unable to bind to microtubules. To resolve this problem, White and Lauring proposed a dual mode of microtubule interaction [6]. According to their view, human spastin's N-terminal MTBD might help positioning tubulin's tail such that the pore loop is able to attack the C-terminus of tubulin. Once properly engaged, the pore might function similar to that of ClpA or ClpB, and exert mechanical pulling-forces on tubulin's C-terminus.

The fact that the MTBD of human spastin is only loosely tethered to the microtubule surface, and that it is not conserved in *Drosophila* makes it difficult to explain , specific interactions of MTBD and microtubule E-hook. The fact that spastin and katanin show similar diffusion coefficients despite different microtubule affinities suggest that the energy barrier that has to be overcome from one E-hook to the next is low.

In the context of the functional construct (GFP-Δ227 HsSpastin), however, we observed tight microtubule binding in the presence of AMPPNP. There are two ways to explain the change of affinity: Possibly, AMPPNP changes the conformation in a way that promotes binding via the pore-loop region, or the molecule oligomerizes and therefore forms multiple bridges to the microtubule. While we cannot exclude the first possibility, our data supports the second alternative. In kymographs, the intensity of the fluorescent severing enzymes was clearly increased under ATP-γS and AMPPNP conditions. The dissociation rates of GFP spastin and GFP katanin were reduced 2.5 to 5-fold (ATP-γS), or even to a rate that was not measurable (AMPPNP). The observation under ATP-γS conditions was supported by co-sedimentation assays, where a significantly higher affinity was observed. In addition, the stoichiometry of severing enzyme per tubulin dimer was increased approximately two- (spastin) or threefold (katanin). The stoichiometry, however, does not necessarily reflect the microscopic assembly state, which could, for example, be one katanin hexamer bound to two tubulin dimers.

The stabilizing effect of microtubules on hexamer formation was also seen in EM images. Although the spastin E442Q mutant can be driven into the hexameric form in the absence of microtubules, these rings are not very stable [8]. Hexameric rings are only visible in EM at high protein concentrations and after quick fixation. In this study, we saw hexameric rings of this mutant in microtubule bundling assays. We were able to obtain these structures at a concentration approximately three orders of magnitude lower.

Interestingly, microtubule binding not only led to oligomerization and stabilization, but, vice versa, the coiled coil spastin dimer mimicked the microtubule-bound state. For this construct, the enzymatic activity was as high in the absence of microtubules as in their presence, suggesting that the interaction with microtubules facilitates the formation of a dimer. The assembly pathway of AAA proteins has not been studied in much detail, but it is very likely that it involves the sequential addition of intermediate oligomers (monomers, dimers, trimers or tetramers) to monomers or existing partial assemblies. The formation of a spastin dimer would be a critical step in this process. Curiously, published cross-linking experiments on *Arabidopsis* katanin suggested the presence of a trimeric intermediate, suggesting that the assembly of AAA proteins does not follow a conserved pathway [32].

Ross and co-workers proposed that diffusion might serve to guide katanin to the ends of microtubules from where it is able to depolymerize the filament (Supporting Movie S4 and [12]). If this is true it does not apply to spastin, which in our hands never showed depolymerizing activity. Still, loose tethering of severing enzymes to the surface of microtubules and diffusion along the filament might be part of a scanning mechanism for favorable sites of mechanistic attack. Our data is consistent with the following model: Spastin monomers bind to microtubules via electrostatic interactions. The loosely bound protein diffuses along the length of the filament, which increases the probability of diffusional encounters of subunits. Once the critical step of dimerization has been overcome, the assembly of hexamers occurs quickly. Hexamers are able to exert a mechanical force on the microtubule lattice that is based on the action of the AAA domain. The MTBD is unlikely to contribute a significant part of the force.

An interesting aspect is the comparison to motor proteins such as kinesin and myosin. For these types of proteins, it has been suggested that the initial interaction with the filament is accomplished by unspecific charged interactions, while the force-producing binding state is tight, highly specific and based on a combination of hydrophobic, van-der-Waals and entropic interactions [41], [42]. This concept seems to be invalid for severing enzymes. Spastin lacking the microtubule binding domain does not bind to a detectable degree to microtubules, katanin in a hugely weaker way. Interestingly, for katanin the binding affinity to subtilisin-treated microtubules is not zero, indicating more than one binding mode. To identify the parts involved in force production, it might be helpful to use this observation.

## Supporting Information

Figure S1 **Steady state ATPase Activities of GFP Δ227 HsSpastin.** The figure shows the ATP- and microtubule-dependence of the GFP spastin construct used for microtubule interaction assays in two replicates. The maximal turnover rates and the activation properties are indistinguishable from those without the N-terminal GFP fusion [8].
(TIF)

Figure S2 **Spastin Concentration-Dependence of Severing.** Severing of microtubules required a minimum concentration of spastin. Severing did not occur without spastin, without ATP, with AMPNPP, or in the presence of the E442Q mutant and 2 mM ATP. The term pre-severing rate indicates that the duration of the lag phase before microtubule breaking occurred was used [8].
(TIF)

Figure S3 **M.s.d trace and analysis of GFP Δ227-HsSpastin in the presence of 1 mM ATP.** Panel A shows the sequence of positions of the tracked particle (in x/y pixels), panel B the calculated m.s.d. trace (red crosses, left axis) and the derived diffusion coefficient (red line, right axis) assuming a one-dimensional diffusion. All local diffusion coefficients of all traces (n = 30) were used for the histograms shown in Figures 2 and 3.
(TIF)

Figure S4 **Kymographs of GFP Spastin Diffusion in Dependence of the Nucleotide.**
(TIF)

Figure S5 **Kymographs of GFP Katanin Diffusion in Dependence of the Nucleotide.**
(TIF)

Movie S1 **Movie S1 shows GFP-Δ227 HsSpastin (green) moving along AlexaFluor 555 microtubules (red).** The movie plays in real time, the height of the canvas is 13,3 µm.
(MP4)

Movie S2 **Movie S2 shows GFP-katanin p60 (green) moving along AlexaFluor 555 microtubules (red).** The movie plays in real time, the width of the canvas is 25.1 µm.
(MP4)

Movie S3 **Movie S3 shows GFP-Δ227 HsSpastin in green channel, severing microtubules attached to the cover slip.** After an incubation time (termed pre-severing time, [1]), microtubules are severed rapidly. The time is given in min∶sec, the field of view is 80 µm. The characteristics did not differ measurably from the GFP-free variant.
(MOV)

Movie S4 **Movie S4 shows the depolymerizing activity of HsKatanin.** The field of view is 80×80 µm, the time stamps indicate minutes. Alexa Fluor 555 microtubules were incubated with 5 nM unlabeled full-length katanin in the presence of 1 mM ATP.
(AVI)
