# Supplementary figures and images for: Spastin's Microtubule-Binding Properties and Comparison to Katanin
Source: PLoS One. 2012 Dec 13;7(12):e50161. doi: 10.1371/journal.pone.0050161 (PMC3521757; doi:10.1371/journal.pone.0050161)

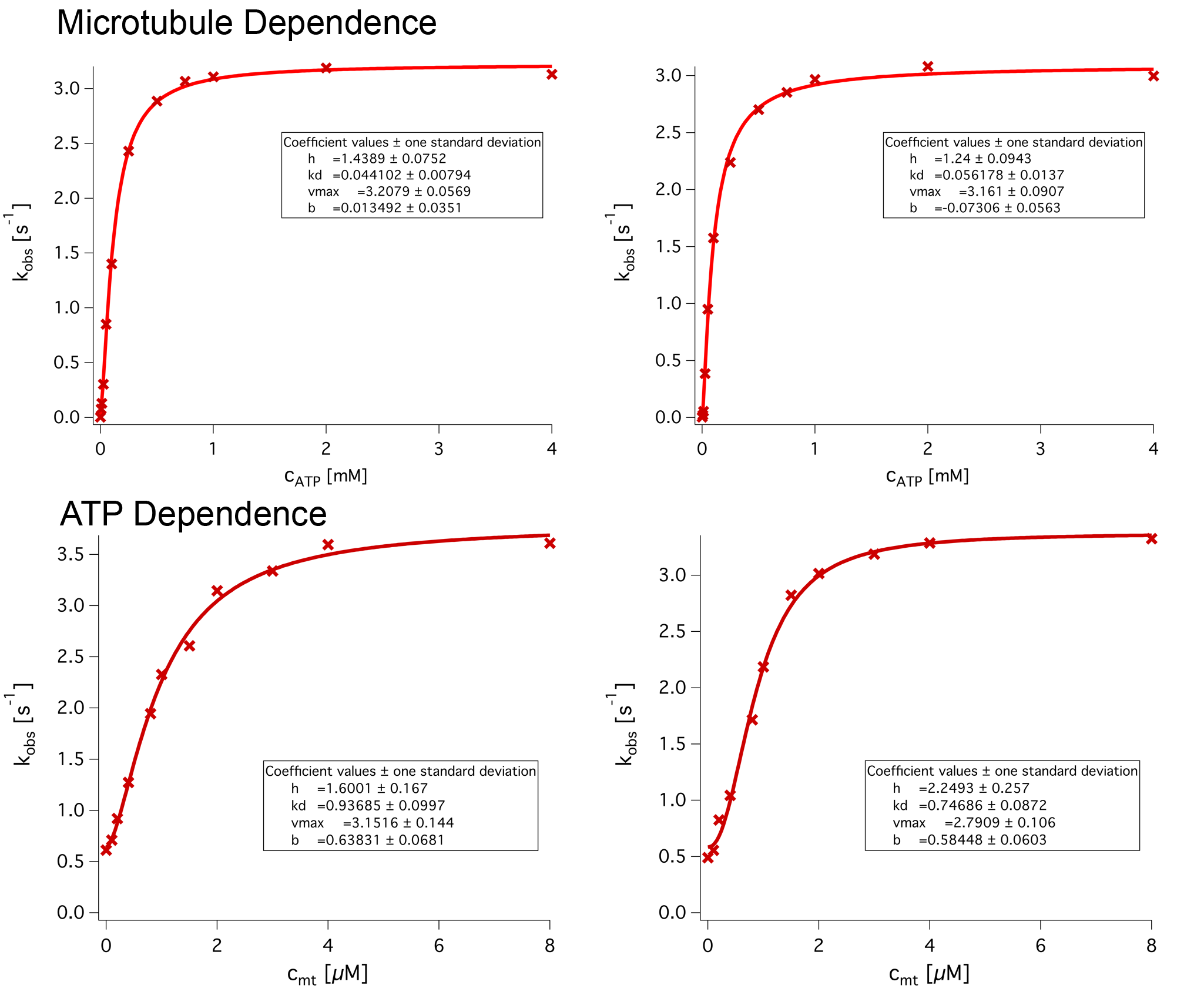

Supplement: Figure S1 — Steady state ATPase Activities of GFP Δ227 HsSpastin. The figure shows the ATP- and microtubule-dependence of the GFP spastin construct used for microtubule interaction assays in two replicates. The maximal turnover rates and the activation properties are indistinguishable from those without the N-terminal GFP fusion [8]. (TIF) [file pone.0050161.s001.tif]

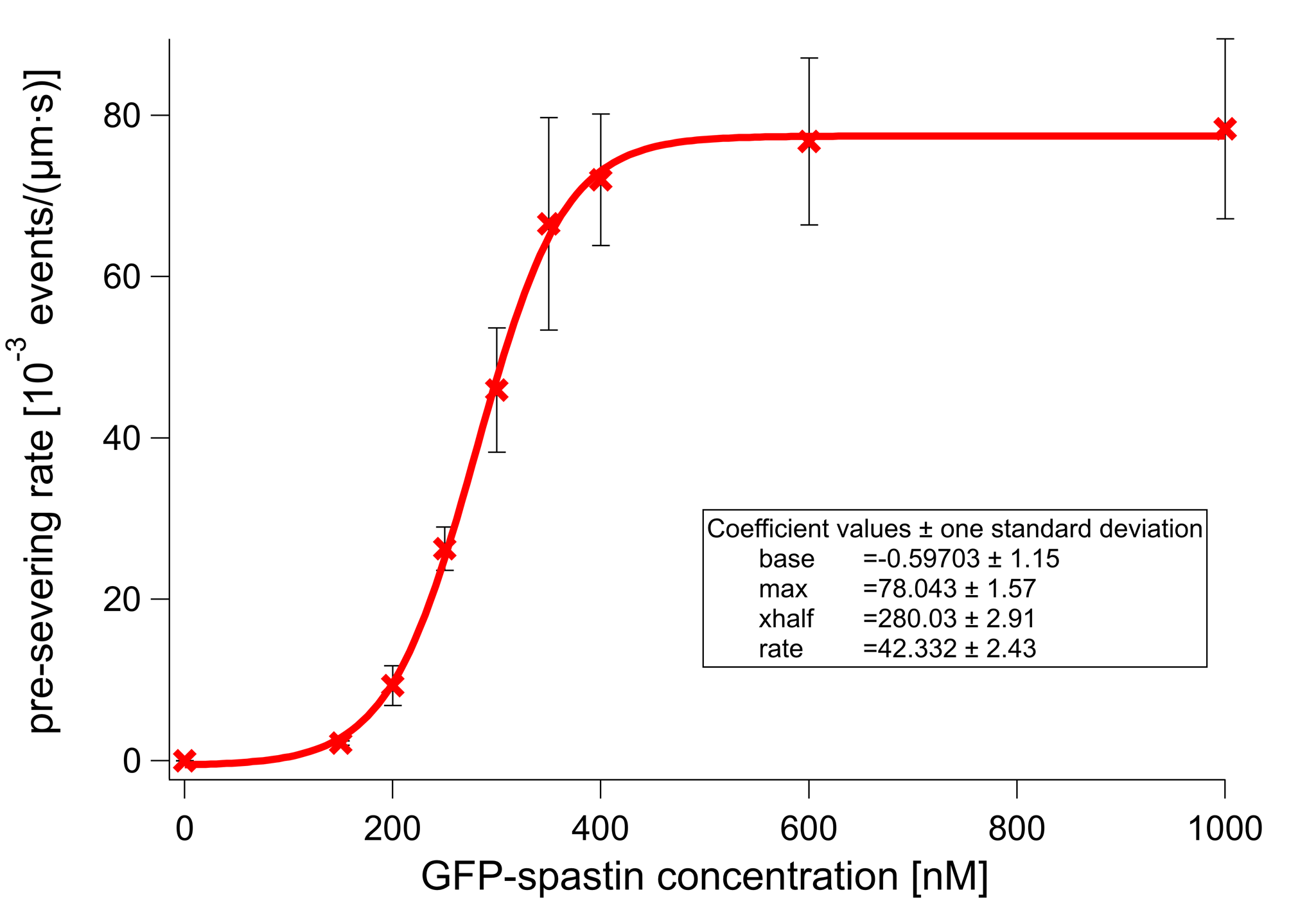

Supplement: Figure S2 — Spastin Concentration-Dependence of Severing. Severing of microtubules required a minimum concentration of spastin. Severing did not occur without spastin, without ATP, with AMPNPP, or in the presence of the E442Q mutant and 2 mM ATP. The term pre-severing rate indicates that the duration of the lag phase before microtubule breaking occurred was used [8]. (TIF) [file pone.0050161.s002.tif]

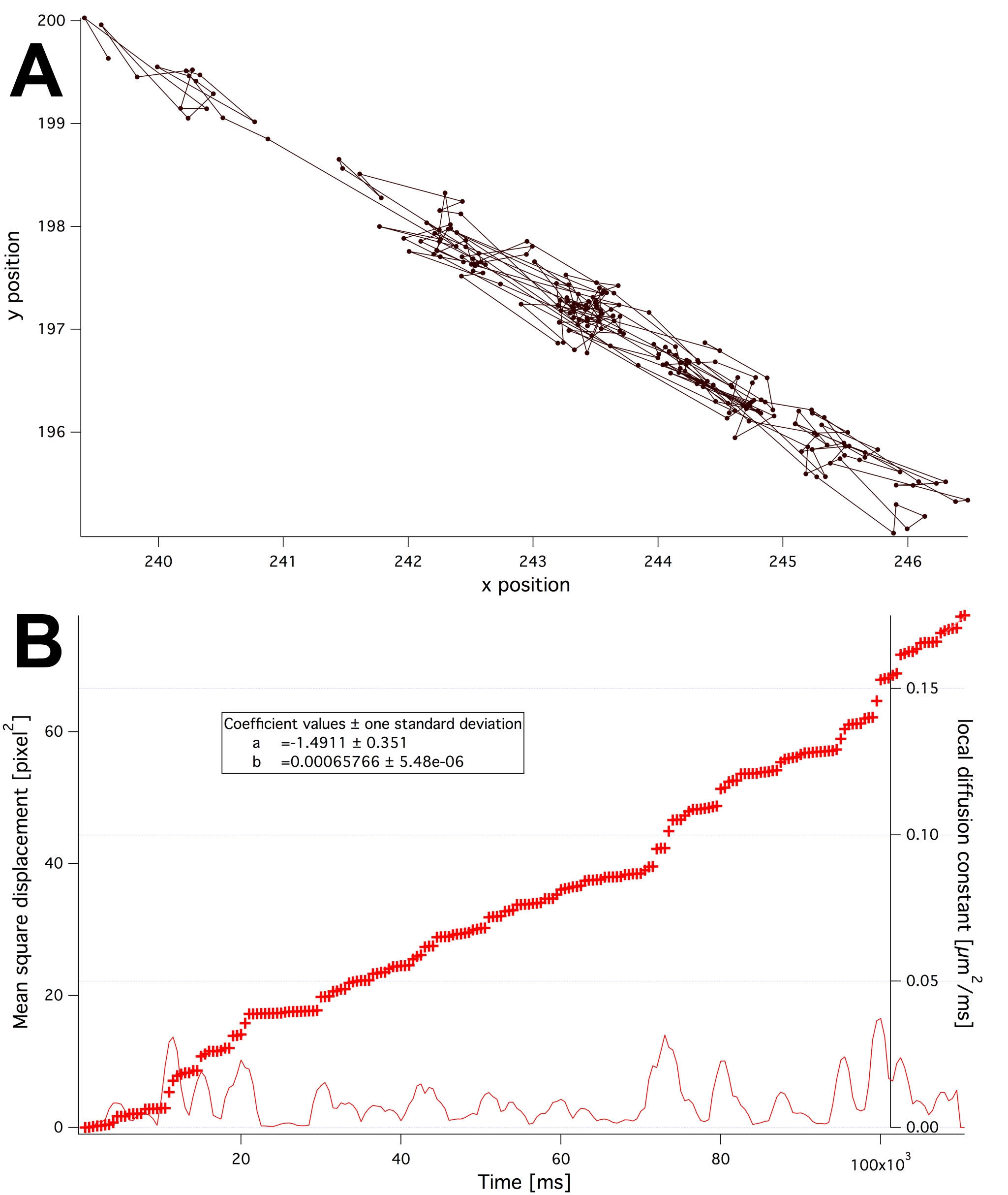

Supplement: Figure S3 — M.s.d trace and analysis of GFP Δ227-HsSpastin in the presence of 1 mM ATP. Panel A shows the sequence of positions of the tracked particle (in x/y pixels), panel B the calculated m.s.d. trace (red crosses, left axis) and the derived diffusion coefficient (red line, right axis) assuming a one-dimensional diffusion. All local diffusion coefficients of all traces (n = 30) were used for the histograms shown in Figures 2 and 3. (TIF) [file pone.0050161.s003.tif]

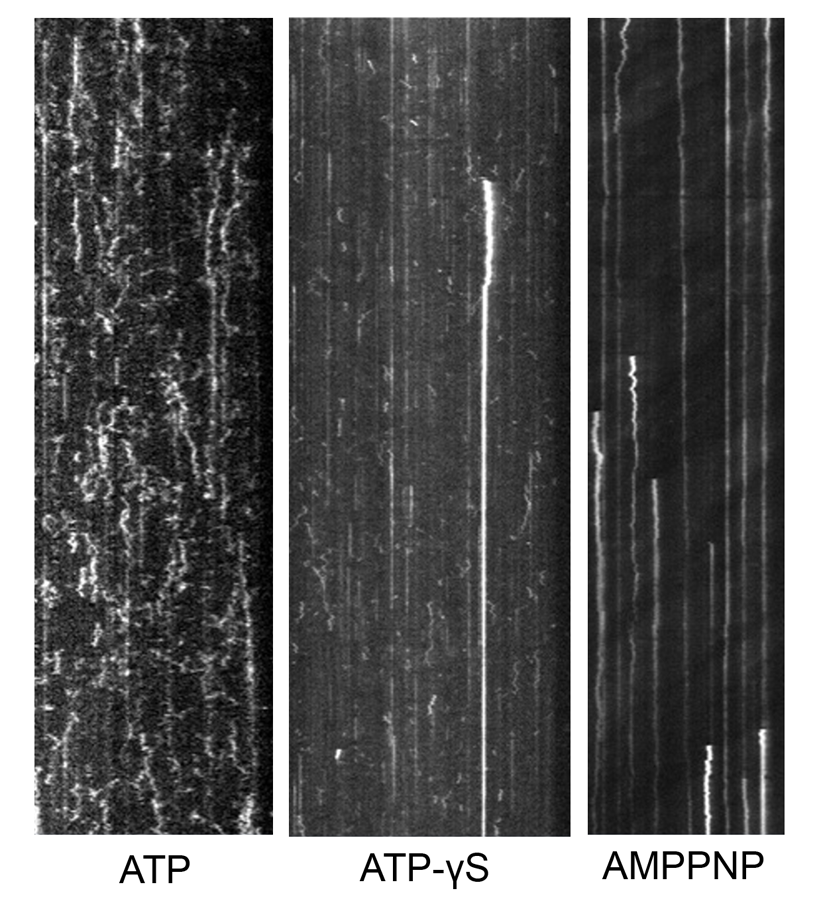

Supplement: Figure S4 — Kymographs of GFP Spastin Diffusion in Dependence of the Nucleotide. (TIF) [file pone.0050161.s004.tif]

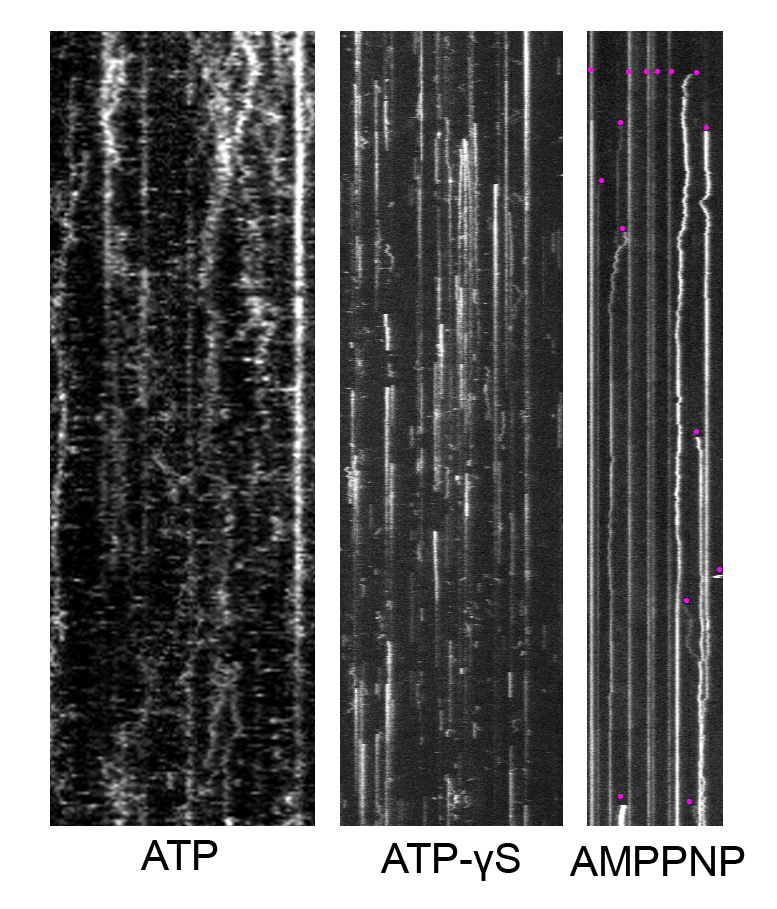

Supplement: Figure S5 — Kymographs of GFP Katanin Diffusion in Dependence of the Nucleotide. (TIF) [file pone.0050161.s005.tif]
